# Curved carbon-plated shoe may further reduce forefoot loads compared to flat plate during running

**DOI:** 10.1038/s41598-024-64177-3

**Published:** 2024-06-08

**Authors:** Yang Song, Xuanzhen Cen, Dong Sun, Kovács Bálint, Yan Wang, Hairong Chen, Shunxiang Gao, István Bíró, Ming Zhang, Yaodong Gu

**Affiliations:** 1 Research Academy of Medicine Combining Sports, Ningbo No.2 Hospital, Ningbo, China; 2 Department of Biomedical Engineering, Faculty of Engineering, The Hong Kong Polytechnic University, Hong Kong, China; 3 Doctoral School on Safety and Security Sciences, Óbuda University, Budapest, Hungary; 4 Faculty of Engineering, University of Szeged, Szeged, Hungary; 5 Faculty of Sports Science, Ningbo University, Ningbo, China; 6 Department of Kinesiology, Hungarian University of Sports Science, Budapest, Hungary

**Keywords:** Musculoskeletal system, Computational models

## Abstract

Using a curved carbon-fiber plate (CFP) in running shoes may offer notable performance benefit over flat plates, yet there is a lack of research exploring the influence of CFP geometry on internal foot loading during running. The objective of this study was to investigate the effects of CFP mechanical characteristics on forefoot biomechanics in terms of plantar pressure, bone stress distribution, and contact force transmission during a simulated impact peak moment in forefoot strike running. We employed a finite element model of the foot-shoe system, wherein various CFP configurations, including three stiffnesses (stiff, stiffer, and stiffest) and two shapes (flat plate (FCFP) and curved plate (CCFP)), were integrated into the shoe sole. Comparing the shoes with no CFP (NCFP) to those with CFP, we consistently observed a reduction in peak forefoot plantar pressure with increasing CFP stiffness. This decrease in pressure was even more notable in a CCFP demonstrating a further reduction in peak pressure ranging from 5.51 to 12.62%, compared to FCFP models. Both FCFP and CCFP designs had a negligible impact on reducing the maximum stress experienced by the 2nd and 3rd metatarsals. However, they greatly influenced the stress distribution in other metatarsal bones. These CFP designs seem to optimize the load transfer pathway, enabling a more uniform force transmission by mainly reducing contact force on the medial columns (the first three rays, measuring 0.333 times body weight for FCFP and 0.335 for CCFP in stiffest condition, compared to 0.373 in NCFP). We concluded that employing a curved CFP in running shoes could be more beneficial from an injury prevention perspective by inducing less peak pressure under the metatarsal heads while not worsening their stress state compared to flat plates.

## Introduction

The shoe longitudinal bending stiffness (LBS) is a footwear feature that has received less attention compared to other shoe characteristics (e.g., midsole cushioning)^1^. However, a lot more attention have drawn to this topic now due to its recognized potential to enhance running performance^2,3^. Generally, this improvement is achieved with the use of a stiff carbon-fiber plate (CFP) inserted along the length of the midsole, which has been reported to be able to optimize the metatarsophalangeal energetics, alter the cost of muscular contraction, and redistribute lower extremity joint work^4–6^. A recent systematic review and meta-analysis revealed that increasing LBS using CFP led to a notable improvement in running economy (RE) up to 3.15% when careful control was maintained over footwear mass^7^.

Prolonged running activities are well-documented to cause tissue damage and material deterioration due to the cyclical submaximal loading nature. If left unaddressed, these issues may eventually lead to foot injuries and subsequent pain^8–11^. To mitigate the risk of running-related foot injuries, it is crucial to consider design features in running shoes that can modify tissue damage accumulation. One potential solution that has been proposed is the use of CFP footwear, which may help relieve painful forefoot syndromes by offloading this area^12^. However, the effectiveness of CFP footwear may vary depending on its design features. For instance, Flores et al.^13^ suggested that a high-loaded CFP, positioned just below the insole, could result in lower perceived comfort and increased plantar pressure under the forefoot. To investigate this further, our previous study examined the effects of different CFP modifications on load changes in plantar tissue and metatarsal bones^14^. The results aligned well with the assumption of Flores et al.^13^, and we also found that, compared to high-loaded CFP, low-loaded conditions (positioned just above the outsole) effectively reduced forefoot plantar pressure and exhibited a gradual reduction in peak metatarsal stress as the stiffness increases. Taking these findings altogether, it is important to emphasize that a wrong choice of CFP design could potentially increase the risk of foot injuries during running.

In a study conducted in 2019, Farina et al. assessed the effect of CFP shapes, including flat, moderate curve, and extreme curve, on metatarsophalangeal (MTP) joint biomechanics^15^. Their findings indicated that the absence of CFP led to the highest energy dissipation at the MTP joint, while the extremely curved CFP (CCFP) exhibited the lowest energy loss. Subsequently, Rodrigo-Carranza et al.^7^ concluded in their review that footwear with increased LBS using CCFP improved RE by 3.45%, while studies using a flat CFP (FCFP) slightly improved RE (0.19%). To date, most studies have focused on FCFP, but the evidence presented above suggests that using CCFP in distance running shoes may offer significant metabolic and performance benefits compared to FCFP. On the other hand, no studies to the best of our knowledge have explored the effects of CFP shapes on internal foot mechanics during running. When optimal running performance is the goal, it is crucial to first uncover the potential injury risks associated with the use of CFP footwear.

The use of musculoskeletal models and simulations has become crucial in the footwear industry, as it allows for fast prediction of how runners’ tissue loading may respond to a specific footwear feature modification^16–18^. In this study we aimed to use the established 3D foot-shoe coupled finite element (FE) model to further determine the effects of CFP stiffness and shape on forefoot plantar pressure, metatarsal stress distribution, and MTP joint force transmission at the impact peak during forefoot strike (FFS) running. Based on the findings of our previous simulation^14^, it was hypothesized that the stiffest CCFP would contribute to the lowest forefoot loads than other conditions.

## Results

### Plantar pressure

**Figure 1:**
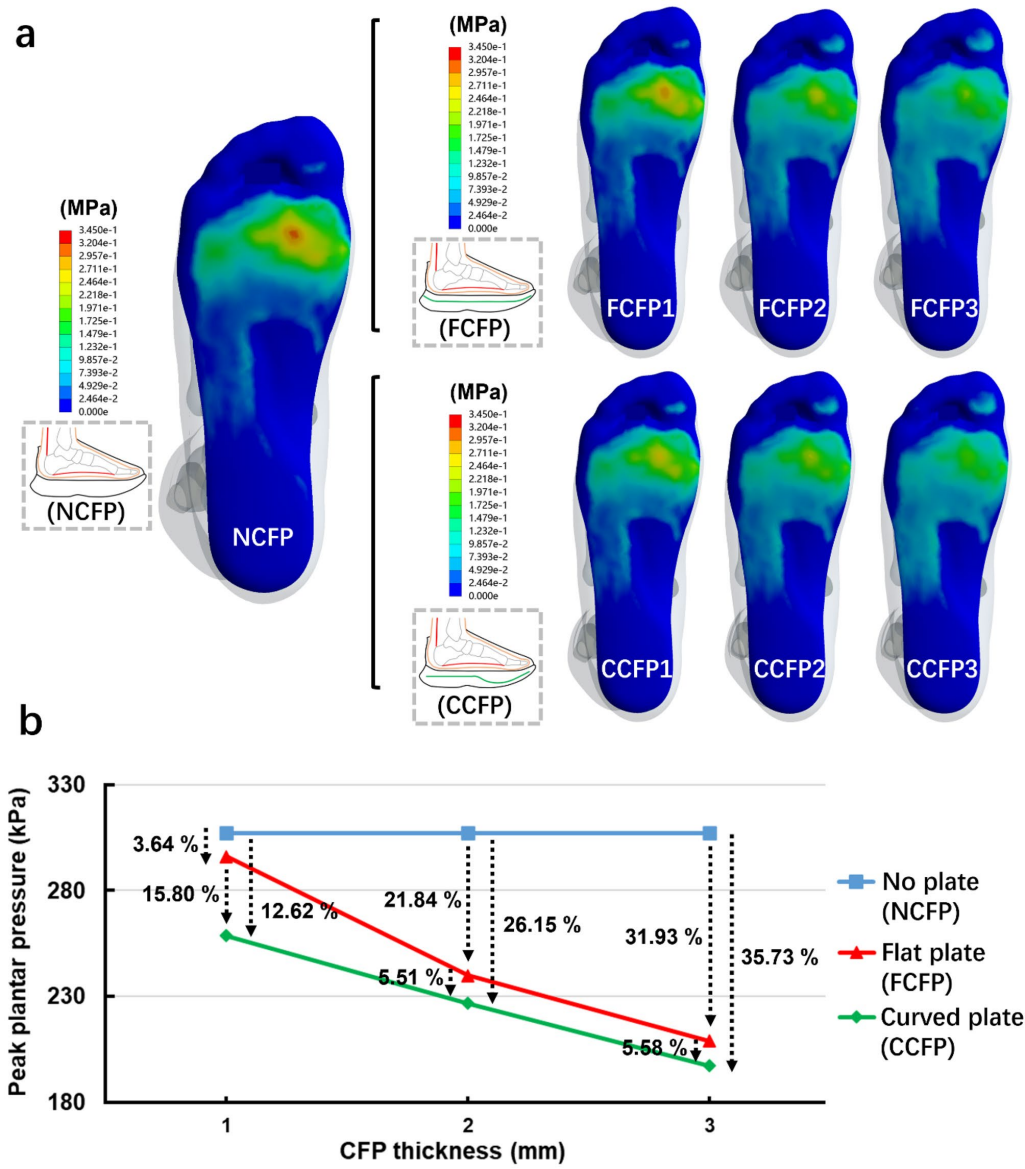
Comparison of plantar pressure in foot-shoe models with respect to different CFP shapes and stiffnesses at the impact peak instant during FFS running. (**a**) Depicts the results of the finite element analysis while (**b**) shows the peak plantar pressure values of each condition.

The peak plantar pressure was located beneath the medial forefoot region (second and third metatarsal shafts) in all models, while the high pressure ‘spot’ gradually disappeared due to increased stiffness of CFP (Fig. 1a). The peak pressure reduction curve (Fig. 1b) shows that compared to NCFP, peak plantar pressure was reduced by 3.64% (FCFP1), 21.84% (FCFP2), and 31.93% (FCFP3) in FCFP models, and 15.80% (CCFP1), 26.15% (CCFP2), and 35.73% (CCFP3) in CCFP models, respectively. When comparing the difference of various CFP shapes on plantar pressure, CCFP models further reduced the peak values by 12.62% (CCFP1), 5.51% (CCFP2), and 5.58% (CCFP3), respectively, compared to those of FCFP. Overall, the peak pressure had a maximum reduction of 35.73% at the CCFP3 condition.

### Bone stress

As shown in Table 1, the peak stress (von Mises) in the 1st, 4th, and 5th metatarsal bones gradually decreased to a maximum by 11.55%, 36.19%, and 42.64%, respectively, due to the FCFP condition, and by 9.69%, 34.62%, and 43.15%, respectively, due to the CCFP condition compared to NCFP condition. The 2nd and 3rd metatarsals showed noticeably greater stress than the other metatarsals, but the peak values were not notably affected due to the use of CFP, except for the FCFP1 case where peak stress in the 2nd metatarsal bone was slightly increased by 4.51%, while it decreased by 8.52% in the 3rd metatarsal.

**Table 1:** Peak von Mises stress (MPa) of the metatarsal bones in foot-shoe models with respect to different CFP shapes and stiffnesses at the impact peak instant during FFS running.

Variables	NCFP	FCFP1	FCFP2	FCFP3	CCFP1	CCFP2	CCFP3
Metatarsal 1	4.85	5.26 8.45% (↑)	4.57 5.77% (↓)	4.29 11.55% (↓)	4.77 1.65% (–)	4.57 5.77% (↓)	4.38 9.69% (↓)
Metatarsal 2	8.87	9.27 4.51% (↑)	8.99 1.35% (–)	8.81 0.68% (–)	9.09 2.48% (–)	8.97 1.13% (–)	8.94 0.79% (–)
Metatarsal 3	8.92	8.16 8.52% (↓)	9.17 2.80% (–)	8.85 0.78% (–)	8.67 2.80% (–)	9.07 1.68% (–)	9.07 1.68% (–)
Metatarsal 4	5.72	5.62 1.75% (–)	4.37 23.60% (↓)	3.65 36.19% (↓)	5.01 12.41% (↓)	4.30 24.83% (↓)	3.74 34.62% (↓)
Metatarsal 5	3.87	3.97 2.58% (–)	2.82 27.13% (↓)	2.22 42.64% (↓)	3.51 9.30% (↓)	2.77 28.42% (↓)	2.20 43.15% (↓)

(↑) indicates increased stress in different shoe conditions relative to the NCFP condition; (↓) indicates decreased stress in different shoe conditions relative to the NCFP condition; (–) indicates less notable stress change in different shoe conditions relative to the NCFP condition.

### Contact force transmission

**Figure 2:**
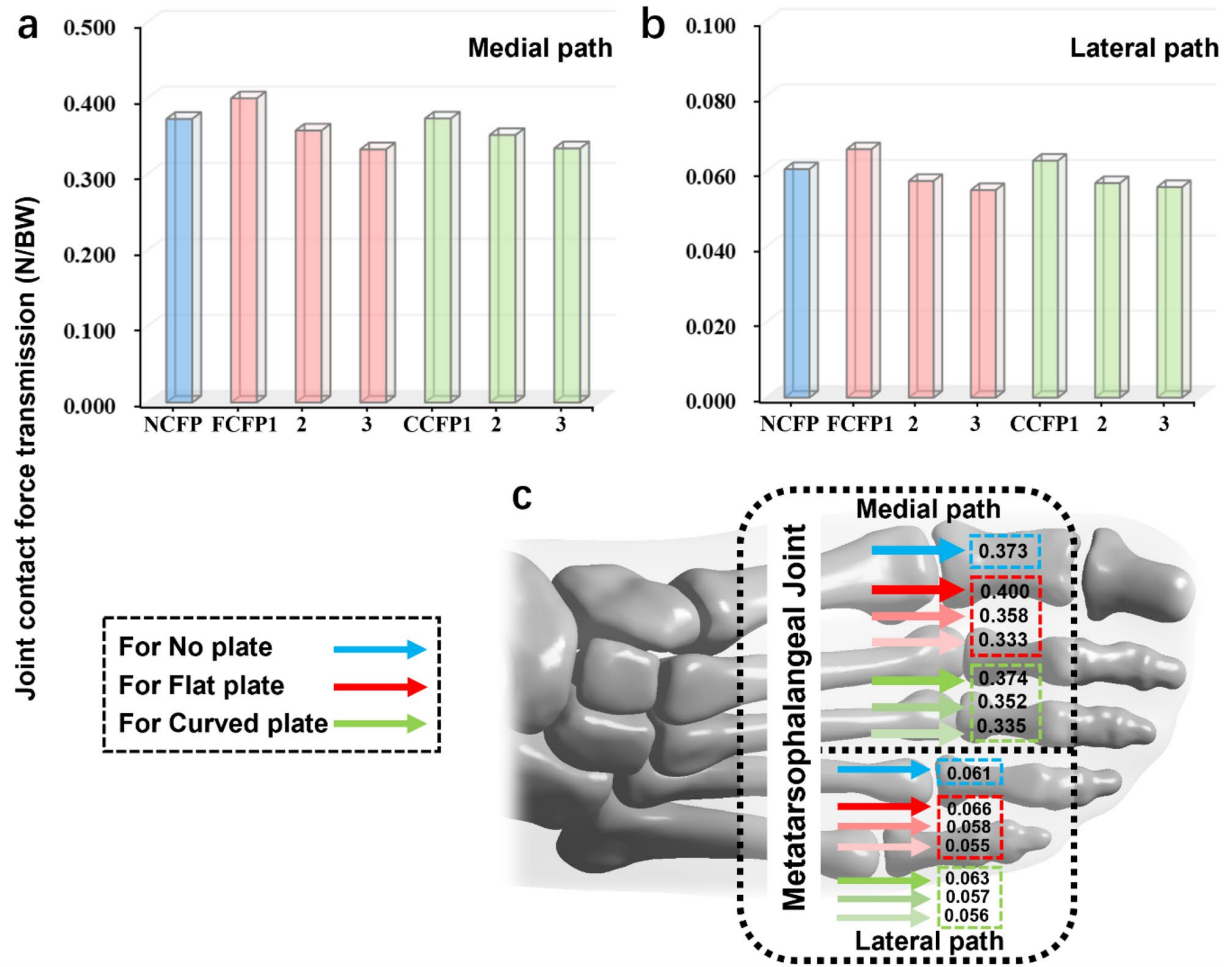
Comparison of MTP joint contact force transmission in foot-shoe models with respect to different CFP shapes and stiffnesses at the impact peak instant during FFS running, (**a**) Medial path of the MTP joint contact force transmission; (**b**) Lateral path of the MTP joint contact force transmission; (**c**) Anatomical schematic illustration of the MTP joint contact force transmission. Force is depicted in terms of times of body weight. Blue arrows are for foot-shoe model without CFP, red arrows are for foot-shoe models with FCFP, and green ones are for foot-shoe models with CCFP. The gradation in coloration represents the variations of CFP thickness. Specifically, the lighter the shading, the greater the thickness of the CFP.

There was a general trend that the force transferred through the MTP joint decreased as CFP stiffness increased (Fig. 2a). As shown in Fig. 2b, in the medial path, about 0.373 times body weight was transmitted through this joint in the NCFP condition, and this decreased to 0.333- and 0.335-times body weight in the FCFP3 and CCFP3 conditions, respectively. Similarly, the force transferred through the lateral path reduced to 0.055- and 0.056-times body weight in the FCFP3 and CCFP3 conditions compared to that of the NCFP condition (0.061). Additionally, a small deviation between the foot-shoe models should be noted. Compared to the NCFP model, the force transmission increased to 0.4 and 0.066 times of the body weight in the FCFP1 model in medial and lateral ways, respectively.

## Discussion

Altering the footwear LBS with the goal of reducing injury risk is gaining momentum. The present study utilized the established FE model of the foot and footwear with sensitivity analysis. Through this analysis, we determined the effects of geometrical variations in CFP, including stiffnesses and shapes, on peak plantar pressure under the forefoot, stress states within the metatarsal bones, and force transmission at the MTP joint.

Plantar pressure is a widely used tool in biomechanical practice as it may provide information about internal foot loading during movement^23^. In alignment with our previous research^14^, the peak plantar pressure under the forefoot consistently decreased when the CFP stiffness increased, eventually contributing to a more even pressure distribution with no obvious high-pressure ‘spot’ over the forefoot plantar surface (Fig. 1a). Notably, the decrease in plantar pressure was more obvious when the shape changes of the CFP were also considered. Our results showed that, in comparison to the FCFP counterparts, the CCFP models exhibited a further reduction in pressure ranging from 5.51 to 12.62% (Fig. 1b), which clearly demonstrated the role of curvature in CFP design. It has been previously reported that the pressure under the forefoot was greatly increased after long-distance running. This increased pressure, if left untreated, may lead to foot pain and running-related overuse foot disorders, as proposed in the previous research^8,24,25^. In this regard, it is indicated based on the findings of this study that a curved CFP in running shoe has the potential to further modify the magnitude of tissue loading and, consequently, may reduce the risk of overuse injuries. Regarding the underlying rationale for an optimal CFP design to maximize pressure reduction, it was previously demonstrated that the FCFP reduces forefoot pressure mainly by dispersing impact forces and limiting MTP joint movement to allow the forefoot to make flat and broad contact with the ground^12^. Thus, the observation of further reduction in peak plantar pressure in this study suggests that CCFP could have better effects in the above two aspects. This was also corroborated by the more even contact pressure observed compared to corresponding FCFP conditions (Fig. 1a). Nevertheless, given the absence of biomechanical research directly comparing plantar pressure among running shoes with different CFP shapes, a confirmation of these theoretical analyses is warranted.

The 2nd and 3rd metatarsal bones are shown to be susceptible to stress fractures, with fractures of the 2nd metatarsal being particularly common and problematic among runners^26,27^. In our study, these two bones exhibited significantly higher von Mises stress compared to the other metatarsals (Table 1), aligning with their clinical vulnerability. However, both the FCFP and CCFP designs appeared to have limited impact on the stress states within these two bones. This suggests that the pressure relief provided to plantar tissues through the use of an optimal CFP may not have as pronounced an effect on the internal loading of the foot as previously assumed^12^. This finding, to some extent, aligns with the results reported by Chen et al.^28^, who observed similar effects in therapeutic insole and metatarsal pad design. It may also lend support to the notion that external force should not be automatically considered representative of internal loading^27,29^. Nevertheless, we did observe a gradual stress reduction trend in the 1st, 4th, and 5th metatarsals (Table 1), indicating that the CFP structure still has a certain effect on offloading metatarsals. Furthermore, our contact force analysis revealed more obvious changes in load transfer along the medial pathways, with a further decrease observed with the use of CFP (Fig. 2). Overall, this scenario may suggest a shift in the load transfer pathway toward more uniform force transmission, potentially leading to reduced foot pain in the medial columns during running. However, all these findings should be further validated by longitudinal epidemiological evidence.

While this study represents the initial exploration of internal foot mechanics and associated injury risks in novel CFP footwear, it is important to acknowledge its inherent limitations. It has been previously reported that the optimal footwear LBS is dependent on various factors, such as running speed and body weight^30,31^. Thus, the single-case design of this study may restrict the generalizability of our research findings to a broader population. Additionally, several assumptions were made in our model, including simplifications in the structural representation of foot bones and ligaments, the use of isotropic linear elastic material properties, and uniform biomechanical input for finite element simulations across all footwear conditions. Given this limitation, the results of this study are intended to offer qualitative insights into the biomechanical effects of CFP from a theoretical standpoint, rather than providing an exact representation of this specific type of footwear. Another limitation pertains to the footwear design itself. Due to constraints related to the prototype running shoe's midsole thickness and structural design, we did not perform a gradient simulation analysis for CFP structures with varying curvatures (such as moderate and extreme curvature CFPs). Meanwhile, Frederick recently proposed the concept of Advanced Footwear Technology (AFT), which specifically refers to a performance-enhancing footwear technology that combines lightweight, resilient midsole foams with rigid moderators and pronounced rocker profiles in the sole^32^. Therefore, it would also be valuable to further investigate the interactive effects of these AFT components on running-related injury risks, which could provide comprehensive guidance for shoe design and optimization.

## Conclusions

Based on our results we can conclude that a running shoe equipped with a CCFP may offer greater potential for overuse injury prevention. Such a design leads to reduced peak pressure under the forefoot without notably impacting the stress state of the metatarsal bones, as compared to the NCFP and FCFP conditions. Employing a CFP with appropriate stiffness, in general, appears to redirect the load transfer pathway toward a more evenly distributed force transmission, potentially mitigating the risk of overuse injuries during long-distance running. The information provided in this study can serve as a baseline for the design and optimization of carbon-plated running shoes.

## Materials and methods

### Participants

This study was performed in compliance with the declaration of Helsinki and ethical approval was granted by Ningbo University Human Subject Ethics Subcommittee (reference number: RAGH20221013). The participant (male, age: 28 years, height: 175 cm, mass: 70 kg, running experience: 5 years) who volunteered for this study was fully informed of the experimental procedures and provided the written consent form. This study consisted of two main parts. Firstly, participants underwent computed tomography (CT) scanning to collect the medical images of foot and shoe, which was then used to reconstruct 3D solid models. Secondly, participants underwent lab-based gait experiments to obtain loading conditions for the FE analysis and to validate the simulation results.

### Running shoe models

For this simulation, the 3D anatomically detailed foot-shoe model generated in our previous study was adopted^14^. The foot model consists of 20 distinct bone segments, 66 ligaments, and 5 plantar fasciae embedded in a volume of encapsulated foot soft tissue. The shoe model includes the upper part and sole components of the running shoe we used in this study. The shoe size was US 41, and the heel-to-toe drop was 8 mm.

**Figure 3:**
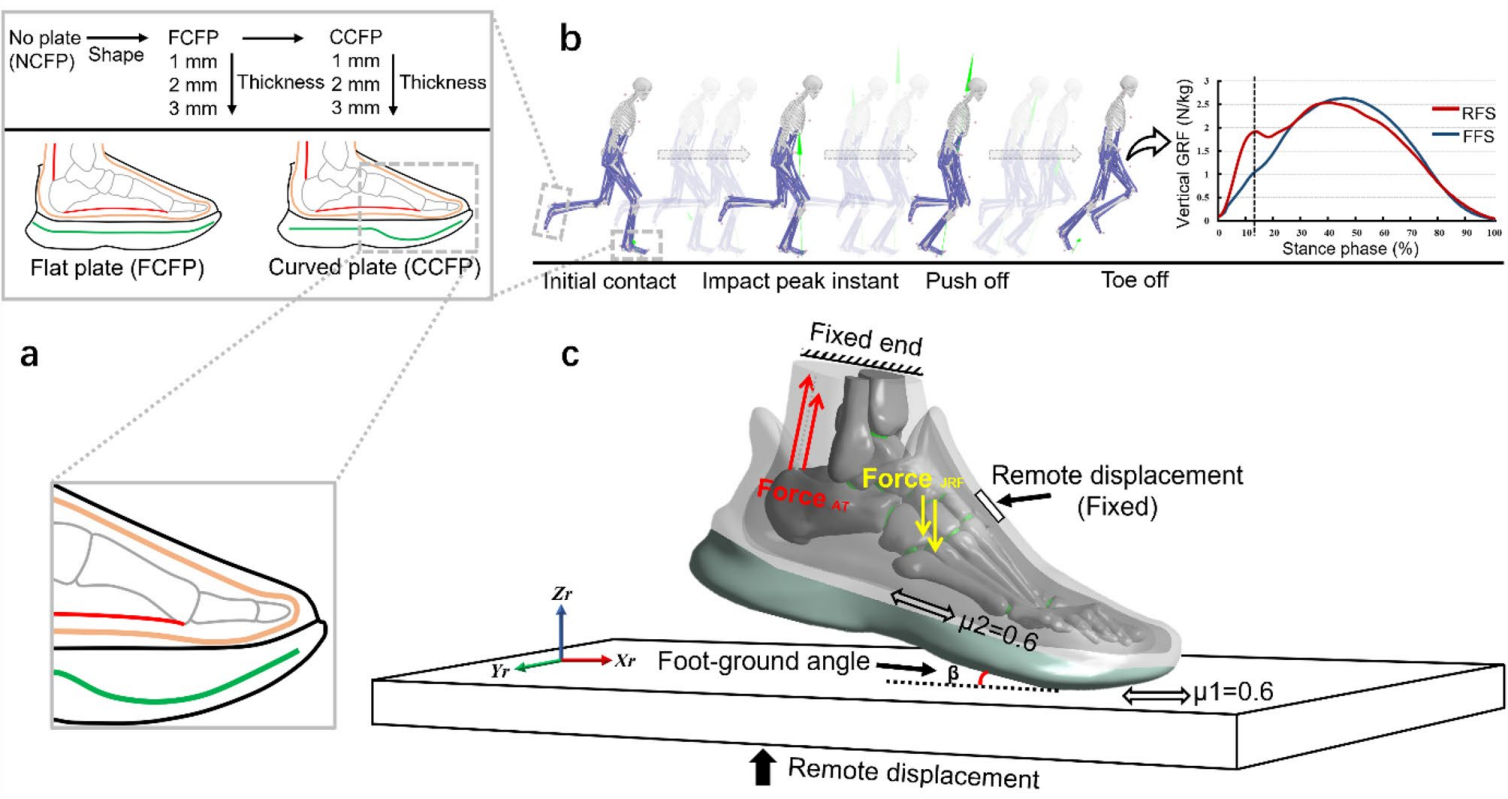
(**a**) Configurations of the different foot-shoe models with respect to different CFP shapes and thicknesses, including no CFP (NCFP, stiffness: 2.19 N/mm), 1 mm-flat CFP (FCFP1, stiffness: 6.25 N/mm), 2 mm-flat CFP (FCFP2, stiffness: 16.36 N/mm), 3 mm-flat CFP (FCFP3, stiffness: 46.25 N/mm), 1 mm-curved CFP (CCFP1, stiffness: 6.25 N/mm), 2 mm-curved CFP (CCFP2, stiffness: 16.36 N/mm), 3 mm-curved CFP (CCFP3, stiffness: 46.25 N/mm); (**b**) Subject-specific musculoskeletal model and simulation of the forefoot running gait; (**c**) Boundary and loading conditions for the finite element analyses. The LBS of the plate was calculated by simulating the flexion mechanical test^19^.

Running shoe models with FCFP and CCFP were further created using computer-aided design software (SolidWorks, Dassault Systèmes, Paris, France). For FCFP, we placed the plate between the midsole and outsole of the shoe, keeping it away from the foot. The location of CFP within the shoe sole is justified by our previous study, demonstrating that this position can effectively reduce forefoot pressure without increased metatarsal stress compared to the no CFP (NCFP) shoe^14^. For CCFP, we have maximized its curvature close to the MTP joint while maintaining the midsole structure of the prototype running shoe. The rest of the CCFP characteristics is the same as the FCFP. To investigate the impact of CFP stiffness on foot biomechanics, we used three different plate thicknesses. Namely, the original plate thickness (1 mm) was increased to 2 mm and 3 mm, referring to stiffer and stiffest scenarios, respectively. Meanwhile, to maintain consistent LBS of the two shapes, the thickness of CCFP was slightly adjusted with the method used by Fu et al.^19^. In total, the FE simulation incorporated 7 distinct design combinations as shown in Fig. 3a.

The material properties for the foot-shoe model were established based on prior simulation settings^16^. Detailed information about the material properties of the FE model is shown in Table 2. The meshing strategy employed hexahedral elements for the ground plate and tetrahedral solid elements for all other components. Typically, a global element size of 3.5 mm was employed for the bone structures, while a 5.0 mm size was used for the soft tissue, shoe, and plate components. To enhance mesh quality, virtual topology techniques were applied to adapt and refine the surface mesh for each component. Moreover, localized mesh refinement was implemented in regions with intricate geometries to improve analysis precision. Additionally, a mesh convergence analysis was carried out, guided by forefoot plantar pressure, to strike a balance between model accuracy and computational resource optimization.

**Table 2:** Element types and material properties of the model components.

Model component	Element type	Young’s modulus E (MPa)	Poisson’s ratio ν
Bone	Tetrahedral solid	7300	0.30
Soft tissue	Tetrahedral solid	1.15	0.49
Ligament	Tension-only spring	–	–
Plantar fascia	Tension-only spring	–	–
Shoe upper	Tetrahedral solid	11.76	0.35
Shoe sole	Tetrahedral solid	2.739	0.35
Carbon fiber plate	Tetrahedral solid	33,000	0.40
Ground plate	Hexahedral solid	17,000	0.10

### Boundary and loading conditions

The simulation focused on the landing impact peak instant during FFS running. During rearfoot strike (RFS) running, a distinctive first peak can be observed on the ground reaction force (GRF) curve and it can be used as the impact peak instant^20^. This timepoint relative to the stance in percentage can be used to locate the same timepoint relative to the stance on a GRF curve of FFS which was used in our simulations (Fig. 3b)^21^. During the experiment, the participant completed 5 FFS trials at a speed of 3.33 m/s on a runway while wearing the control running shoe. The trial that was closest to the target speed and had the step falling within the force plate area was selected for further analysis.

As shown in Fig. 3c, the proximal cross-section surfaces of the fibula, tibia, and bulk soft tissue as well as the shoe tongue were fixed. To simulate the impact peak instant, the ground plate was rotated to the corresponding foot–ground angle (7.11°) in the sagittal plane and limited to move only in the vertical direction. We implemented the measured variable into the FE simulation as the vertical GRF (712 N) from gait analysis and the estimated Achilles tendon force (1744 N) and MTP joint contact force (548 N) from the musculoskeletal model calculated in OpenSim (National Center for Simulation in Rehabilitation Research, Stanford, USA). Details of the experimental procedure and data calculation were explained in Ref.^14^.

The simulations were computed in Ansys Workbench 2022 (ANSYS, Inc., United States) with the standard static solver. The results included foot plantar pressure and metatarsal bone stress, as well as contact force transmission (in terms of times of body weight) across the MTP joint, which was separated into medial (the first three rays) and lateral path (the last two rays) of load transfers^22^. The peak values of the outcome variables were quantitatively compared among the 7 conditions.

### Experimental validation

The foot-shoe FE model has been validated by comparing the predicted foot and outsole pressure with the measurements under balanced standing and FFS running conditions^14,17^. During the experimental trials, two pressure measurement systems were employed—the Pedar Insole system (Novel GmbH, Munich, Germany) was used to capture plantar pressure values, while the Footscan system (RSscan International, Olen, Belgium) was utilized to obtain outsole pressure data. For the validation analysis, both the plantar and outsole areas were divided into several specific regions. The plantar area was segmented into the first metatarsal, second metatarsal, third metatarsal, fourth and fifth metatarsals, as well as the hallux. The outsole area was divided into the medial forefoot and lateral forefoot sections. The intraclass correlation coefficient (ICC) analysis showed excellent agreement between experimental measurements and predictions (ICC score = 0.97), and the Bland–Altman plot presented a mean offset of 2.4 kPa without statistical significance.

## Data Availability

The datasets used and/or analyzed during the current study are available from the corresponding author on reasonable request.
